# Some ethical dilemmas

**DOI:** 10.12669/pjms.296.4395

**Published:** 2013

**Authors:** Shabih Zaidi

Imamia Medics International (www.imamiamedics.org), is a global family of doctors, dentists, nurses, paramedics, registered as an NGO in NJ, with a permanent seat in UNO. It is doing many activities, but mainly the charitable services in many countries, and health related education. Their flag ship is the American University of Barbados, school of medicine. (www.aubmed.org)

Recently IMI held its 6th. International conference in Sheraton, Toronto, Canada. More than 500 people from 20 countries participated. It was a powerful, resourceful and academically highly profitable conference. IMI-Canada deserves all the accolades. The plenary session was dedicated to Islamic Jurisprudence and Moral dilemmas. As expected, it generated huge interest, as matters like definition of death, and the time of ensoulment, surrogacy and organ transplants to and from non Muslims etc were some of the issues highlighted.

Learned Ulema and eminent physicians shared the debate. Some issues were resolved others continue to pose dilemmas. One such issue is the ageless debate on the definition of death. Is it the Somatic or the Brain death, which should be the point of no return. Apart from the final ceremonies etc, one major dilemma is that of timing organ harvesting.

According to the scientists it is the Brain death which is the final call. An amazing series of MRI scans were shown by a speaker, which displayed the gradual disappearance of electric activity from the cortex to the brain stem during the dying stages. It was as if you were looking at a power house gradually and systematically losing power, ending in a total black out.

The religious definition of death, Ulema insist, is the somatic death also described as *Qalb Ki Mot.* That definition raises many a question, particularly the very basic principle of cardiopulmonary resuscitation (CPR), or basic life support (BLS). If the person is considered dead with the functional stoppage of the heart, then where does the medical profession stand in terms of resuscitation?

IMI has developed certain guidelines called the Toronto declaration, which still needs further discussion, before it is posted on their web page. So last week the matter of *Qalb Ki Mot *was discussed by this writer in a seminar in Manchester.

The word Qalb and the word F'uad are used in Quran in lieu of mind, Intellect, and intelligence, not the brain per se. In fact the holy book does not mention the word brain or mind as such. It always refers to Qalb as the source of intellect, intelligence i.e. mind. (The reference is available on the net. *Al-Imran -151; Qaf- 33, & 37; Tawba-117).*

The word F'uad also has been used in Quran with the meaning of mind, in Suare-Ibrahim-37, al-Nahl-78, al-Sajda-9, al-Humaza-7, al-Anam-110, al-Hud-120.

So the question needs to be answered for many reasons. What is death? Is it the somatic death or the brain death, that should be taken the irrevocable point of returning to our Khaliq, as declared in the book...*'Ina Lillah wa Ina eleh rajaoon'.*

